# Higher frequency but similar recurrence rate of uveitis episodes in axial spondylarthritis compared to psoriatic arthritis. A multicentre retrospective study

**DOI:** 10.1007/s00296-023-05424-0

**Published:** 2023-08-23

**Authors:** Nikolaos Kougkas, Konstantina Magiouf, Chrysoula G. Gialouri, Gerasimos Evangelatos, Maria Pappa, Aikaterini Dimouli, Alexios Iliopoulos, Anastasios Karmanakos, Theodoros Dimitroulas, Maria G. Tektonidou, Petros P. Sfikakis, George E. Fragoulis

**Affiliations:** 1 Department of Rheumatology, Ippokration Hospital, Thessaloniki, Greece; 2 Joint Academic Rheumatology Program, First Department of Propedeutic and Internal Medicine, National and Kapodistrian University of Athens, Athens, Greece; 3 Joint Academic Rheumatology Program, Clinical Immunology, Rheumatology unit, Second Department of Medicine and Laboratory, National and Kapodistrian University of Athens, Athens, Greece; 4 Department of Rheumatology, 417 Army Share Found Hospital (NIMTS), Athens, Greece; 5 Department of Rheumatology, “Evangelismos” General Hospital, Athens, Greece

**Keywords:** Uveitis, Spondyloarthritis, Psoriatic arthritis

## Abstract

**Background/Objective:**

Data on risk factors predicting uveitis development in spondyloarthritis (SpA) is scarce. Our aim was to examine associations between demographic, clinical and/or laboratory characteristics of SpA with the occurrence and the course of uveitis, including ocular damage and recurrence rate.

**Methods:**

Characteristics (at disease diagnosis and ever-present) from axSpA and Psoriatic arthritis (PsA) patients followed in 3 tertiary rheumatology-clinics were retrospectively recorded. Comparisons were made between patients with and without uveitis, as well as between those with uveitis-rate [episodes/year] above the median uveitis-rate in the whole cohort (“recurrent”-uveitis) and the remaining uveitis patients (“non-recurrent uveitis”). In multivariable models, age, gender and variables significantly different in univariate analyses were included.

**Results:**

264 axSpA and 369 PsA patients were enrolled. In axSpA, uveitis occurred in 11.7% and was associated with HLA-B27 (OR = 4.15, 95%CI 1.16–14.80, *p* = 0.028) and ever-present peripheral arthritis (OR = 3.05 (1.10–8.41, *p* = 0.031). In contrast, uveitis in PsA occurred only in 2.7% of patients and was associated with SpA family-history (OR = 6.35 (1.29–31.27), *p* = 0.023) axial disease at diagnosis (OR = 5.61 [1.01–28.69], *p* = 0.038) and disease duration (OR = 1.12 [1.04–1.21], *p* = 0.004). Median uveitis recurrence rate was comparable between axSpA and PsA (0.205 and 0.285 episodes/year, respectively). No associations were found between recurrent uveitis and demographic/clinical/laboratory characteristics. Ocular damage (e.g. synechiae) was seen in 16.1% of axSpA and 30% of PsA patients, all of them with recurrent uveitis.

**Conclusion:**

Uveitis occurred more commonly in axSpA than in PsA patients, while uveitis recurrence rate was similar. Permanent ocular damage may occur more often in PsA than axSpA.

**Supplementary Information:**

The online version contains supplementary material available at 10.1007/s00296-023-05424-0.

## Introduction

The term spondyloarthropathy (SpA) encompasses a group of chronic inflammatory disorders of the joints, including psoriatic arthritis (PsA), peripheral SpA, enteropathic arthritis and axial SpA (axSpA) which incorporates non-radiographic axial SpA (nraxSpA) and ankylosing spondylitis (AS) [1]. The sacroiliac joints, spine, peripheral joints, tendons and entheses are the most commonly affected sites in SpA [2]. However, people living with SpA frequently develop extra-articular manifestations including uveitis, psoriasis, and inflammatory bowel disease [3].

Up to one-third of individuals with SpA experience uveitis, which is usually anterior and unilateral and represents the most common extra-articular feature [4, 5]. The prevalence of uveitis varies across the different forms of SpA, with evidence supporting that uveitis is more common in AxSpA compared to PsA [4]. Despite that uveitis in SpA has been associated with some demographic (e.g. male gender) and clinical or laboratory characteristics (e.g. HLA-B27) (8–10), data are still scarce [4].

The clinical course of uveitis varies, and inadequate or inappropriate treatment can lead to accumulating damage and possibly vision loss [6]. According to the Standardization of Uveitis Nomenclature (SUN) Working Group Anatomic Classification of Uveitis, acute uveitis has a sudden onset and a short duration, while the term “recurrent” should be used to describe repeated episodes of uveitis separated by periods of inactivity without treatment that last at least 3 months [7]. In general, recurrent anterior uveitis increases the risk of ocular complications, such as cataract, glaucoma, band keratopathy, and posterior synechiae [8, 9]. Thus far, it is unknown whether specific SpA features are related to recurrent uveitis. This is partly related also to the lack of definition for recurrent uveitis in the setting of SpA.

In this retrospective study we aimed to examine possible associations between demographic, clinical and laboratory characteristics and the occurrence and the course (including recurrence and ocular damage) of uveitis, in SpA patients. In this context, we also estimated the recurrence rate of uveitis in our real-world SpA cohort.

## Patients and methods

We screened medical records from 3 tertiary centers in Greece, of all axSpA (fulfilling the ASAS classification criteria for axSpA [10]) andPsA (fulfilling CASPAR criteria [11] patients attended, at least two times, the rheumatology outpatients and inpatients clinics from January 2018 to January 2023. Patients with peripheral SpA (other than PsA) as well as patients who were less than 18 years old at the time of diagnosis were excluded [12]. The following data were extracted: occurrence of uveitis verified by ophthalmologist, at any time from the diagnosis of SpA and thereafter, demographics (age, gender, current smoking, body mass index, family history [first degree relative] of SpA, laboratory (HLA-B27 positivity), clinical features at disease onset and ever present (namely: peripheral arthritis, axial involvement for PsA [relevant symptomatology accompanied by x-rays or magnetic resonance imaging-MRI], psoriasis, enthesitis, dactylitis, nail disease, inflammatory bowel disease [confirmed by colonoscopy]), as well as treatment history of SpA. The time of uveitis diagnosis, as well as the number and the type (unilateral/bilateral) of uveitis episodes were recorded. Ocular damage (synechiae, blindness, glaucoma, cataract), if any, was also documented. We calculated for each patient the rate of uveitis episodes as the number of uveitis episodes per years of follow-up since the diagnosis of the index disease (i.e. axSpA or PsA).

For both axSpA and PsA, clinical, laboratory and demographic features were compared between patients with and without uveitis. Moreover, patients were considered to have “recurrent uveitis” when their rate of uveitis episodes was above the median uveitis episodes rate of the entire cohort (separately for axSpA and PsA). Characteristics of patients with “recurrent uveitis” were compared to those without (“non-recurrent uveitis”). An additional analysis was also performed, comparing characteristics of patients who displayed only one episode of uveitis to those with more than one episode.

The current study was conducted according to the Declaration of Helsinki. The study was approved by the Scientific councils of “Laiko” (No: 780-21, date: 4-4-2021), “NIMTS” (No: 196-19, date: 5-11-2021) and “Ippokratio” (No: 55195, date: 1-11-2022). All patients provided written informed consent.

Statistics.

Categorical and numerical features were compared with two-sided Fisher’s and Mann–Whitney tests, respectively. Kolmogorov–Smirnov test was used to check for normality. Binomial logistic regression was performed, having as dependent variable the occurrence of uveitis and age, gender, and variables that were statistically different in univariate analyses, as independent variables. Results were expressed as odds ratios (ORs). Statistical significance is considered for *p-*values less than 0.05. Regarding sample size calculation, this was not performed a priori in our study, as we included all patients that were actively followed-up in our departments. However, using the Daniel formula with confidence intervals 95% and margin of error 5%, adequate number was calculated to be 246 patients for axSpA (estimated uveitis rate: 20%) and 59 patients for PsA (estimated uveitis rate: 4%) [13]. GraphPad Prism 5.00 (GraphPad Software, Inc., USA) and SPSS 24.0 (SPSS software, USA) were used.

## Results

### Cohort description

In total, 264 patients with axSpA and 369 patients with PsA were enrolled. AxSpA patients were 43.5% females with a mean (SD) age of 49.5 (14.1) years, while individuals with PsA were 54.4% females with a mean (SD) age of 54.5 (13.0) years. Further characteristics of patients enrolled in the study are depicted in Table 1.

**Table 1:** Demographic, laboratory, and clinical characteristics of axSpA and PsA patients

Feature	axSpA (n = 264)	PsA (n = 369)
Demographics		
Age (years), mean (SD)	49.5 (14.1)	54.5 (13.0)
Female gender, n (%)	115 (43.5)	201 (54.4)
Follow-up time, years, mean (SD)	15.7 (14.1)	13.8 (9.4)
BMI, mean (SD)	27.9 (15.4)	28.4 (5.8)
Family history of SpA, n (%)	26/224 (11.6)	48/328 (14.6)
Smoking (current), n (%)	97/237 (40.9)	122/341 (35.7)
HLA-B27, n (%)	117/193 (60.6)	25/132 (18.9)
Clinical (Ever)		
Peripheral arthritis, n (%)	101 (38.3)	344 (93.2)
Enthesitis, n (%)	50 (18.9)	104 (28.1)
Dactylitis, n (%)	11 (4.1)	81 (21.9)
Bowel involvement^∞^, n (%)	27 (10.2)	18 (4.8)
Uveitis, n (%)	31 (11.7)	10 (2.7)
Axial-disease, n (%)	N/A	115 (31.1)
Psoriasis, n (%)	27 (10.2)	N/A

*axSpA* axial spondyloarthritis, *BMI* body mass index, *n* number, *PsA* psoriatic arthritis, *SD* standard deviation, * clinical plus imaging (x-ray or magnetic resonance) evidence, ∞ inflammatory bowel disease confirmed by colonoscopy

### Association of uveitis occurrence with demographic, clinical and laboratory features

In patients with axSpA, uveitis occurred in 31 of 264 (11.7%) at a mean (SD) 6.2 (4.3) years after the diagnosis of the index disease. Of them, 21 were already on treatment with bDMARDs (eleven with monoclonal antibodies against TNF, 8 with etanercept and one with secukinumab) at the time of first episode. Uveitis was always affecting the anterior chamber and was unilateral in half (16/31, 51.6%) of the cases. Uveitis occurrence was associated with HLA-B27 positivity (*p* = 0.005) and with ever-present peripheral arthritis (*p* = 0.05) (Table 2). In the multivariable analysis, statistical significance remained for both HLA-B27 [OR (95% CI) = 4.15 (1.16–14.80), *p* = 0.028] and peripheral arthritis [OR (95% CI) = 3.05 (1.10–8.41), *p* = 0.031]. Of note, when we ran another model in which disease duration (marginally significant in the univariate analysis) was included, results did not change [for HLA-B-27; OR (95% CI) = 4.14 (1.11–15.43), *p* = 0.035; and for peripheral arthritis OR (95% CI) = 3.05 (1.10–8.42), *p* = 0.031].

**Table 2:** Comparison of demographic, laboratory, and clinical characteristics between axSpΑ patients who developed uveitis and those who did not

Feature	axSpA		p-value
	Uveitis (n = 31)	Non-uveitis (n = 233)	
Demographics			
Age (years), mean (SD)	52.3 (15.2)	49.2 (13.9)	0.245
Female gender, n (%)	14 (45.1)	101 (43.3)	0.849
BMI, mean (SD)	26.0 (3.5)	28.2 (16.5)	0.518
Family history of SpA, n (%)	6/26 (23.0)	20/198 (10.1)	0.093
Smoking (current), n (%)	13/29 (44.8)	84/208 (40.3)	0.689
HLA-B27, n (%)	20/23 (86.9)	97/170 (57.0)	**0.005**
Disease duration, mean (SD)	17.2 (10.8)	15.6 (16.4)	0.059
Clinical at diagnosis			
Peripheral arthritis, n (%)	6/26 (23.0)	52/157 (33.1)	0.368
Enthesitis, n (%)	0/26 (0.0)	17/154 (11.0)	0.138
Dactylitis, n (%)	0/26 (0.0)	8/154 (5.1)	0.604
Bowel involvement^∞^, n (%)	1/26 (3.8)	12/154 (7.7)	0.695
Clinical (ever)			
Peripheral arthritis, n (%)	17 (54.8)	84 (36.1)	**0.050**
Enthesitis, n (%)	8 (25.8)	42 (18.0)	0.329
Dactylitis, n (%)	3 (9.6)	8 (3.4)	0.126
Bowel involvement^∞^, n (%)	1 (3.2)	26 (11.1)	0.220
Psoriasis, n (%)	4 (12.9)	23 (9.8)	0.536

*axSpA* axial spondyloarthritis, *SD* standard deviation, *n* number, ∞ inflammatory bowel disease confirmed by colonoscopy

In patients with PsA, uveitis occurred in only 10/369 (2.7%) of patients after a mean (SD) 4.8 (5.5) years after the diagnosis of the index disease. In 4 of them, uveitis occurred despite they were already on treatment with bDMARDs (2 with monoclonal antibodies against TNF, 1 with etanercept and one with secukinumab). Anterior uveitis was unilateral in most (7/10, 70%) of the cases. Uveitis occurrence was associated with a family history of SpA, (*p* = 0.008), axial disease at diagnosis of index disease (*p* = 0.004), disease duration (*p* = 0.012), as well as with the ever-occurrence of dactylitis (*p* = 0.045), enthesitis (*p* = 0.033) and bowel involvement (*p* = 0.009) (Table 3). In the multivariable analysis, family history of SpA [OR (95% CI) = 6.35 (1.29–31.27, *p* = 0.023], axial disease at diagnosis [OR (95% CI) = 5.61 (1.01–28.69), *p* = 0.038] and disease duration [OR (95% CI) = 1.12 (1.04–1.21), *p* = 0.004] remained statistically significant.

**Table 3:** Comparison of demographic, laboratory, and clinical characteristics between PsA patients who developed uveitis and those who did not

Feature	PsA		p-value
	Uveitis (n = 10)	Non-uveitis (n = 359)	
Demographics			
Age (years), mean (SD)	46.6 (7.5)	54.7 (13.1)	0.089
Female gender, n (%)	7/10 (70.0)	194/359 (54.0)	0.356
BMI, mean (SD)	24.7 (3.2)	28.6 (5.9)	0.053
Family history of SpA, n (%)	5 (50.0)	43/318 (13.5)	**0.008**
Smoking (current), n (%)	5 (50.0)	117/331 (35.3)	0.338
HLA-B27, n (%)	3/7 (42.8)	22/125 (17.6)	0.124
Disease duration, mean (SD)	14.8 (9.2)	9.0 (9.1)	**0.012**
Clinical at diagnosis			
Peripheral arthritis, n (%)	8 (80.0)	231/286 (80.7)	1.000
Enthesitis, n (%)	3 (30.0)	38/329 (11.5)	0.107
Dactylitis, n (%)	1 (10.0)	31/343 (9.0)	1.000
Axial disease*, n (%)	6 (60.0)	57/332 (17.1)	**0.004**
Bowel involvement^∞^, n (%)	0 (00.0)	3/353 (0.8)	1.000
Clinical (ever)			
Peripheral arthritis, n (%)	9 (90.0)	335 (94.3)	0.508
Enthesitis, n (%)	6 (60.0)	98 (27.2)	**0.033**
Dactylitis, n (%)	5 (50.0)	76 (21.1)	**0.045**
Axial disease*, n (%)	6 (60.0)	109 (30.3)	0.076
Bowel involvement^∞^, n (%)	3 (30.0)	15 (4.1)	**0.009**

*PsA* psoriatic arthritis, *SD* standard deviation, *n* number, * clinical plus imaging (x-ray or magnetic resonance) evidence, ∞ inflammatory bowel disease confirmed by colonoscopy

### Recurrent uveitis and ocular damage. Association with demographic, clinical and laboratory features

In axSpA patients 12/31 (38.7%) had only one episode, while the rest 19/31 (61.3%) displayed more than one episode. Median uveitis episodes rate was 0.205 episodes/year. In PsA, only 2/10 (20%) patients had only one episode, while the other eight patients exhibited more than one uveitis episode and the median uveitis episodes was 0.285 episodes/year.

No associations were identified between recurrent uveitis and the above-mentioned demographic, clinical and laboratory characteristics for both disease subsets (Supplementary Tables 1,2). In a sensitivity analysis, we compared axSpA and PsA patients with one episode of uveitis, versus those who exhibited more episodes. In axSpA subgroup, patients with more than one uveitis episodes (compared to those who had only one uveitis episode) displayed more common peripheral arthritis at diagnosis of index disease (*p* = 0.018). This remained significant after adjustment for disease duration (Table 4). For PsA, no associations were identified (Table 5).

**Table 4:** Comparison of demographic, laboratory, and clinical characteristics between axSpA patients who developed 1 episode of uveitis and those who developed > 1 episodes

Feature	axSpA		p-value
	Uveitis (1 episode, n = 12)	Uveitis (> 1 episode, n = 19)	
Demographics			
Age (years), mean (SD)	51.5 (18.4)	52.8 (13.4)	0.827
Female gender, n (%)	6 (50.0)	8 (42.1)	0.724
BMI, mean (SD)	25.4 (3.4)	26.4 (3.7)	0.549
Family history of SpA, n (%)	0/10 (0.0)	6/16 (37.5)	0.053
Smoking (current), n (%)	4/10 (40.0)	9 (47.3)	1.000
HLA-B27, n (%)	7/8 (87.5)	13/15 (86.6)	1.000
Clinical at diagnosis			
Peripheral arthritis, n (%)	5/10 (50.0)	1/16 (6.2)	0.0184
Enthesitis, n (%)	0/10 (0.0)	0/16 (0.0)	1.000
Dactylitis, n (%)	0/10 (0.0)	0/16 (0.0)	1.000
Bowel involvement^∞^, n (%)	1/10 (10.0)	0/16 (0.0)	0.384
Clinical (ever)			
Peripheral arthritis, n (%)	8 (66.6)	9 (47.3)	0.460
Enthesitis, n (%)	4 (33.3)	4 (21.0)	0.675
Dactylitis, n (%)	2 (16.6)	1 (5.2)	0.543
Bowel involvement^∞^, n (%)	1 (8.3)	0 (00.0)	0.387
Psoriasis, n (%)	1 (8.3)	3 (15.7)	1.000

*axSpA* axial spondyloarthritis, *SD* standard deviation, *n* number, ∞ inflammatory bowel disease confirmed by colonoscopy

**Table 5:** Comparison of demographic, laboratory, and clinical characteristics between PsA patients who developed 1 episode of uveitis and those who had > 1 episodes

Feature	PsA		p-value
	Uveitis (1 episode, n = 2)	Uveitis (> 1 episode, n = 8)	
Demographics			
Age (years), mean (SD)	45.0 (16.9)	48.2 (5.4)	0.614
Female gender, n (%)	2 (100)	5 (62.50)	1.000
BMI, mean (SD)	20.7 (3.8)	25.9 (2.2)	NA
Family history of SpA, n (%)	1 (50.0)	4 (50.0)	1.000
Smoking (current), n (%)	1 (50.0)	4 (50.0)	1.000
HLA-B27, n (%)	1 (50.0)	2 (40.0)	1.000
Clinical at diagnosis			
Peripheral arthritis, n (%)	2 (100)	6 (75.0)	1.000
Enthesitis, n (%)	1 (50.0)	2 (25.0)	1.000
Dactylitis, n (%)	0 (0.0)	1 (12.5)	1.000
Axial disease*, n (%)	2 (100)	4 (50.0)	0.466
Bowel involvement^∞^, n (%)	0 (0.0)	0 (0.0)	1.000
Clinical (ever)			
Peripheral arthritis, n (%)	2 (100)	7 (87.5)	1.000
Enthesitis, n (%)	2 (100)	4 (50.0)	0.466
Dactylitis, n (%)	0 (0.0)	5 (62.5)	0.444
Axial disease*, n (%)	2 (100)	4 (50.0)	0.466
Bowel involvement^∞^, n (%)	0 (0.0)	3 (37.5)	1.000

*PsA* psoriatic arthritis, *SD* standard deviation, *n* number, * clinical plus imaging (x-ray or magnetic resonance) evidence, ∞ inflammatory bowel disease confirmed by colonoscopy, *NA* not applicable: comparisons were not concluded due to low number of patients (n = 2) in one category

As regards to the permanent ocular damage (synechiae, blindness, cataract or glaucoma) in these patients, this was seen less commonly in axSpA than in PsA [5/31 (16.1%) versus 3/10 (30%), *p* = 0.378]patients. Of note, all of them displayed recurrent uveitis.

## Discussion

In this retrospective study, we found that uveitis is associated with positivity for HLA-B27 and with ever-present peripheral arthritis in axSpA, and with axial disease at diagnosis, disease duration and family history of SpA in patients with PsA. We also found that in a real world-setting, the median recurrence rate of uveitis is 0.205 (episode/year) for axSpA and 0.285 for PsA.

Uveitis is not uncommon in the setting of SpA. Traditionally it is estimated to occur in about 20–30% of SpA patients throughout the disease course [4, 14–17] with the frequency being higher in axSpA compared to PsA, albeit including the pre-biologic era. In our contemporary cohort, the frequency of uveitis episodes was about 10% for axSpA and 3% for PsA patients. This is in agreement with the largest contemporary study in PsA, so far [18]. For axSpA, It could be suggested that frequency of uveitis in this context tends to decline [4], possibly owe to the introduction of bDMARDs [19]. Uveitis in the setting of SpA is usually acute, anterior and unilateral, as observed in the current study, and is considered a later rather than earlier sequela of the disease [4, 18]. Indeed, we report that the mean (SD) time of uveitis occurrence is 6.2 (4.3) years for axSpA and 4.8 (5.5) years for PsA.

Uveitis has a considerable clinical burden affecting the quality of life and functional ability of patients [18]. In SpA, it usually displays a favorable prognosis with topical treatment with glucocorticoids and/or cycloplegics alone. However, it would be practical if treatments targeting SpA could also prevent uveitis flares. As captured also in the EULAR, GRAPPA and ACR recommendations [20–23], monoclonal antibodies against TNFi are preferred over other b- and ts-DMARDs [20, 21]. Therefore, it would be desirable to identify which patients exhibit higher odds to develop this manifestation.

Various risk factors have been suggested to associate with uveitis in SpA, with HLA-B27 positivity being the most well established in axSpA [14, 17, 24], as it has also been shown in recent large studies [25]. This is also confirmed in our study. Furthermore, in agreement with a previous observational study in AS patients [26], we showed that ever-present peripheral arthritis in patients with axSpA is also associated with uveitis occurrence.

For PsA, Delmás et al. have found an association between uveitis and HLA-B27, while they also report that PsA patients with uveitis have more frequently sacroiliitis on MRI compared to those without, implying that uveitis might be more often encountered in axial-PsA [18]. It has to be noted however, that definition of axial-PsA differs among studies [27]. Interestingly, we found that the presence of axial-disease at diagnosis in patients with PsA is associated with uveitis development. Importantly, we also observed a significant association in PsA patients between family history of SpA and uveitis. This remained statistically significant after adjustments for age, gender and other parameters that were significantly different in univariate analyses. This could be possibly attributed to the close relationship between family history of SpA and HLA-B27 positivity in axSpA [28, 29]. In our multivariable model, when HLA-B27 was inserted as independent variable, positive family history of SpA remained marginally significant parameter (*p* = 0.059). Besides the role of HLA-B27 in PsA remains elusive, it has been reported that HLA-B27 positive axial-PsA patients show similarities with axSpA and perhaps also higher uveitis rates, [30]. It is not irrational to hypothesize that there is an overlap and/or possible misclassification of patients with axSpA as axial-PsA and vice versa*.* Along these lines, it has to be noted that although axSpA and PsA are both classified under the umbrella of SpA and share a lot of clinical manifestations, the extra-articular features as well as the comorbidities occur in different frequencies in these two entities [31]. In terms of pathophysiology it is still unclear to what extent the underlying pathogenetic mechanisms differ, but it seems that IL-23 is a key-regulator in PsA and IL-17 in axSpA [32].

Despite effective treatments for uveitis, recurrent episodes can recur. The concept of “recurrence” is also used as phrasing in the recent EULAR recommendations for axSpA [22]. Relevant definitions are lacking thus far, especially in the context of SpA. Herein, considering the time period during which the uveitis episodes occurred, we defined “recurrence”, when the rate of uveitis episodes was more than 0.205 for axSpA and 0.285 for PsA (which were the median values of uveitis episodes rates in our cohort). Unfortunately, we were not able to detect any demographic, clinical or laboratory features, associated with recurrent uveitis. Of note, peripheral arthritis at the diagnosis of axSpA was associated with more than one episodes of uveitis in these patients.

Anterior uveitis can lead to unfavorable outcomes including synechiae and cataract [14]. This could be related to inadequate treatment, delayed diagnosis or to the natural course of the disease, with patients with chronic uveitis having more often permanent ocular damage [33]. In our cohort, 16.1% of axSpA and 30% of PsA uveitis patients resulted in ocular damage. Interestingly, all patients with irreversible ocular damage displayed recurrent uveitis, underlying the importance of strict control of ocular inflammation.

We acknowledge that our study has certain limitations. Due to the retrospective design of our study, the frequency of uveitis was obtained mainly through medical files in outpatient rheumatology clinics. Second, the study was not designed to detect possible associations between bDMARD treatment received and episodes of uveitis. This has already been addressed by other SpA-studies which have shown that monoclonal antibodies against TNF are more effective compared to IL-17 inhibitors and etanercept [34]. Of note, in our study at the time of first episode, half of the patients were treated with bDMARDs that do not possess efficacy for uveitis (i.e. etanercept, secukinumab), possibly thus affecting our results. Third, patients with peripheral SpA (other than PsA) were not included in our study. This might have led to the exclusion of some patients, however, it helped the homogeneity of our data.Besides, a recent study showed that only a minority of patients suffering from uveitis, was eventually diagnosed with peripheral SpA [35]. Finally, patients in which uveitis preceded the diagnosis of index disease were not included due to insufficient data regarding the diagnosis of uveitis, possibly leading to underestimation of uveitis frequency in the context of SpA [35]. On the other hand, our study has certain strengths. It is one of the largest in the field directly comparing axSpA and PsA patients, identifying novel features (i.e. family history of SpA for PsA and peripheral arthritis for axSpA) associated with uveitis in the setting of PsA. Also, it defines the recurrence rate of uveitis in a real-world study. This might be compared with future studies that will try to define the term “recurrent uveitis” in SpA.

In conclusion, uveitis is more frequent by more than fourfold in axSpA than PsA. Its occurrence associates with specific characteristics like HLA-B27, peripheral arthritis and family history of SpA in axSpA and PsA patients, respectively. Ocular inflammation resulted in ocular damage in about 15–30% of our patients, all of them with recurrent uveitis, underlying the importance of early identification and treatment of these patients.

## Supplementary Information

Below is the link to the electronic supplementary material.

Supplementary file1 (DOCX 32 KB)

## Data Availability

Data are available upon reasonable request to the corresponding author.

## Declarations

### Previous related congress abstract publication

Data presented in this manuscript have not been published in any form so far in abstract congresses (but have been submitted as an abstract in the forthcoming American College of Rheumatology Congress, San Deigo, 2023).

### Open data sharing

Raw data are reasonable upon reasonable request to the corresponding author.
