# Supplementary material for: Higher frequency but similar recurrence rate of uveitis episodes in axial spondylarthritis compared to psoriatic arthritis. A multicentre retrospective study
Source: Rheumatol Int. 2023 Aug 23;43(11):2081–8. doi: 10.1007/s00296-023-05424-0 (PMC10495278; doi:10.1007/s00296-023-05424-0)
Supplement: Supplementary file 1 — Supplementary file1 (DOCX 32 KB) [file 296_2023_5424_MOESM1_ESM.docx]

|  | **axSpA** | |  |
| --- | --- | --- | --- |
| **Feature** | **Recurrent**  **(n= 17)** | **Non-recurrent**  **(n= 14)** | **p-value** |
| **Demographics** |  |  |  |
| Age (years), mean (SD) | 52.7 (14.5) | 51.8 (16.6) | 0.872 |
| Female gender, n (%) | 8 (47.0) | 6 (42.8) | 1.000 |
| BMI, mean (SD) | 26.7 (3.9) | 25.0 (2.9) | 0.253 |
| Family history of SpA, n (%) | 5/15 (33.3) | 1/11 (9.0) | 0.197 |
| Smoking (current), n (%) | 8 (47.0) | 5/12 (41.6) | 1.000 |
| HLA-B27, n (%) | 10/12 (83.3) | 10/11 (90.9) | 1.000 |
| **Clinical at Diagnosis** |  |  |  |
| Peripheral Arthritis, n (%) | 2/16 (12.5) | 4/10 (40.0) | 0.162 |
| Enthesitis, n (%) | 0/16 (00.0) | 0/10 (00.0) | 1.000 |
| Dactylitis, n (%) | 0/16 (00.0) | 0/10 (00.0) | 1.000 |
| Bowel involvement^∞^, n (%) | 0/16 (00.00 | 1/10 (10.0) | 0.384 |
| **Clinical (Ever)** |  |  |  |
| Peripheral Arthritis, n (%) | 7 (41.1) | 10 (71.4) | 0.149 |
| Enthesitis, n (%) | 3 (17.6) | 5 (35.7) | 0.412 |
| Dactylitis, n (%) | 0 (0.0) | 3 (21.4) | 0.081 |
| Bowel involvement^∞^, n (%) | 0 (0.0) | 1 (7.1) | 0.451 |
| Psoriasis, n (%) | 2 (11.7) | 2 (14.2) | 1.000 |

**Supplementary Table-1:** Comparison of demographic, laboratory, and clinical characteristics between axSpA patients who developed recurrent uveitis and those who did not (not-persistent).

axSpA: axial spondyloarthritis, SD: standard deviation, n: number, ∞ inflammatory bowel disease confirmed by colonoscopy.

|  | **PsA** | |  |  |
| --- | --- | --- | --- | --- |
| **Feature** | **Recurrent**  **(n=6)** | **Non-recurrent (n= 4)** | **p-value** |  |
| **Demographics** |  |  |  |  |
| Age (years), mean (SD) | 48.5 (6.1) | 46.2 (10.1) | 0.670 |  |
| Female gender, n (%) | 4 (66.6) | 3 (75.0) | 1.000 |  |
| BMI, mean (SD) | 25.9 (2.2) | 23.3 (4.0) | 0.257 |  |
| Family history of SpA, n (%) | 4 (66.6) | 1 (25.0) | 0.523 |  |
| Smoking (current), n (%) | 4 (66.6) | 1 (25.0) | 0.523 |  |
| HLA-B27, n (%) | 1/3 (33.3) | 2 (50.0) | 1.000 |  |
| **Clinical at Diagnosis** |  |  |  |  |
| Peripheral Arthritis, n (%) | 5 (83.3) | 3 (75.0) | 1.000 |  |
| Enthesitis, n (%) | 2 (33.3) | 1 (25.0) | 1.000 |  |
| Dactylitis, n (%) | 1 (16.6) | 0 (0.0) | 1.000 |  |
| Axial Disease*, n (%) | 3 (50.0) | 3 (75.0) | 0.571 |  |
| Bowel involvement^∞^, n (%) | 0 (0.0) | 0 (0.0) | 1.000 |  |
| **Clinical (Ever)** |  |  |  |  |
| Peripheral Arthritis, n (%) | 6 (100) | 3 (75.0) | 0.400 |  |
| Enthesitis, n (%) | 3 (50.0) | 3 (75.0) | 0.571 |  |
| Dactylitis, n (%) | 3 (50.0) | 2 (50.0) | 1.000 |  |
| Axial Disease*, n (%) | 3 (50.0) | 3 (75.0) | 0.571 |  |
| Bowel involvement^∞^, n (%) | 2 (33.3) | 1 (25.0) | 1.000 |  |

**Supplementary Table-2:** Comparison of demographic, laboratory, and clinical characteristics between PsA patients who developed recurrent uveitis and those who did not (not-persistent).

PsA: psoriatic arthritis, SD: standard deviation, n: number, * clinical plus imaging (x-ray or magnetic resonance) evidence, ∞ inflammatory bowel disease confirmed by colonoscopy.
